# Expert opinions on informational and supportive needs and sources of obtaining information in patients with inflammatory bowel disease: a Delphi consensus study

**DOI:** 10.3389/fpsyg.2023.1224279

**Published:** 2023-09-21

**Authors:** Narges Norouzkhani, Ali Bahari, Javad Shokri Shirvani, Mahbobeh Faramarzi, Saeid Eslami, Hamed Tabesh

**Affiliations:** ^1^Department of Medical Informatics, Faculty of Medicine, Mashhad University of Medical Sciences, Mashhad, Iran; ^2^Department of Internal Medicine, Faculty of Medicine, Mashhad University of Medical Sciences, Mashhad, Iran; ^3^Department of Internal Medicine, Babol University of Medical Sciences, Babol, Iran; ^4^Department of General Courses, Population, Family and Spiritual Health Research Center, Health Research Institute, Babol University of Medical Sciences, Babol, Iran; ^5^Pharmaceutical Research Center, Mashhad University of Medical Sciences, Mashhad, Iran

**Keywords:** inflammatory bowel diseases, needs assessment, informational need, information seeking behavior, consumer health information, supportive needs, psychosocial need, Delphi technique

## Abstract

**Background:**

The present study introduces informational and supportive needs and sources of obtaining information in patients with inflammatory bowel disease (IBD) through a three-round Expert Delphi Consensus Opinions method.

**Methods:**

According to our previous scoping review, important items in the area of informational and supportive needs and sources of obtaining information were elucidated. After omitting duplicates, 56 items in informational needs, 36 items in supportive needs, and 36 items in sources of obtaining information were retrieved. Both open- and close-ended questions were designed for each category in the form of three questionnaires. The questionnaires were sent to selected experts from different specialties. Experts responded to the questions in the first round. Based on the feedback, questions were modified and sent back to the experts in the second round. This procedure was repeated up to the third round.

**Results:**

In the first round, five items from informational needs, one item from supportive needs, and seven items from sources of obtaining information were identified as unimportant and omitted. Moreover, two extra items were proposed by the experts, which were added to the informational needs category. In the second round, seven, three, and seven items from informational needs, supportive needs, and sources of obtaining information were omitted due to the items being unimportant. In the third round, all the included items gained scores equal to or greater than the average and were identified as important. Kendall coordination coefficient W was calculated to be 0.344 for information needs, 0.330 for supportive needs, and 0.325 for sources of obtaining information, indicating a fair level of agreement between experts.

**Conclusions:**

Out of 128 items in the first round, the omission of 30 items and the addition of two items generated a 100-item questionnaire for three sections of informational needs, supportive needs, and sources of obtaining information with a high level of convergence between experts' viewpoints.

## 1. Introduction

The increasing prevalence of inflammatory bowel disease (IBD) in developed and developing countries imposes a significant burden on healthcare systems (Calvet et al., 2014), which has led to an emerging global health concern (Molodecky et al., 2012). IBD mainly appears in two forms: ulcerative colitis (UC) and Crohn's disease (CD). Chronic immune-mediated inflammatory gastrointestinal impairments are the underlying causes of multiple acute life-threatening complications, such as toxic megacolon, sepsis due to penetrating disorder, and thromboembolism (Carter et al., 2004). Although the exact etiology of IBD remains elusive, a complex interaction of genetic (Orholm et al., 1991) and environmental (Danese et al., 2004) factors is found to be responsible for the abnormal activation of the mucosal immune system (Baumgart and Carding, 2007). Both disorders are characterized by periods of remission and active intestinal inflammation, such as diarrhea and abdominal pain, that may even result in hospitalization (Langholz et al., 1994; Munkholm et al., 1995). Additionally, UC and CD increase the risk of colorectal cancer by up to 18% (Eaden et al., 2001). Associated primary sclerosing cholangitis may lead to cholangiocarcinoma. Accordingly, IBD patients are prone to high mortality, either directly or indirectly (Selinger et al., 2013). Because of the chronic nature of these disorders, their unpredictable disease course, their onset at young ages, and the high cost of medical and surgical treatments, they cause social isolation and mood disorders such as depression and anxiety (Sajadinejad et al., 2012; Moradkhani et al., 2013; Williet et al., 2014).

IBD is historically managed in a reactive and crisis-driven mode rather than proactive (Crohn's and Colitis Australia, 2013). Several models of care have been developed for IBD and can be used to overcome certain barriers to quality care. The WHO proposes an integrated approach to improve care quality and avoid disease complications (Jackson and De Cruz, 2019). It is patient-centered and involves patients in service developments; it includes an action plan for follow-up, contains education, incorporates a detailed evaluation of biopsychosocial functioning, and has a dedicated nurse for care coordination (Mikocka-Walus et al., 2012). This approach reduces the frequency of clinic visits, hospitalizations, and polypharmacy, which decreases healthcare costs (Mikocka-Walus et al., 2013).

However, such an integrated model of care is not accessible to all IBD patients, and only large tertiary centers can provide such multidisciplinary care. Another model of care for IBD patients is participatory care, in which patients play a role in the management of the disease. A collaboration is formed between the patient and the physician, while the patient is responsible for driving the healthcare system. Various electronic health tools (Eysenbach and CONSORT-EHEALTH Group, 2011), including web-based platforms, smartphone applications, telemedicine, and decision-support instruments, facilitate the implementation of this model of care. The participatory model promotes patient engagement, augments monitoring of the disease condition, and makes easy earlier intervention (Jackson et al., 2016). Value-based healthcare has recently emerged as a model of care that aims to improve quality in healthcare. It evaluates health outcomes and associated costs at the disease level (van Deen et al., 2015). This model is ultimately designed to overcome hurdles related to care costs (van Deen et al., 2017).

In recent years, the focus of disease management has been on patients rather than their disease. Patients with enhanced knowledge show a higher quality of life and are eager to obtain more information about their disease (Bernstein et al., 2011). Hence, elevating the perception of patients about IBD and its treatment options through care optimization by improving the information provided and augmenting education increased the quality of life and reduced depression and anxiety (Elkjaer et al., 2008). However, educating patients alone is not enough, and self-care strategies also improve disease symptoms, psychological wellbeing, and the use of healthcare resources (Barlow et al., 2010). A study showed that patients who had been trained in self-management care demonstrated higher confidence, had more ability to deal with their condition, experienced fewer hospitalizations, and maintained their quality of life at an appropriate level (Kennedy et al., 2004).

Evidence-based medicine (EBM) uses the best-known findings from current clinical care research diligently and wisely to integrate clinical expertise and manage individual patients (Hohmann et al., 2018). Although EBM is an outstanding approach, it has not yet been sufficiently developed for certain topics with a lack of evidence or uncertainty (Powell, 2003; Keeney et al., 2006). In such circumstances, a consensus opinion of experts is a suitable alternative. One of the available methods in this regard is the Delphi method. In this study, a panel of experts was established without any face-to-face data exchange. Data were collected by distributing sequential questionnaires in at least two rounds. Experts were informed about the feedback from each round in an anonymous way, and finally, an opinion systematically emerged (Hohmann et al., 2018). The advantages of the Delphi method are anonymity, controlled feedback, and statistical group responses (Dalkey and Helmer, 1963; Dalkey, 1969).

Data regarding indices of supportive needs, information needs, and sources of obtaining information for IBD patients are scarce. It is important to elucidate such indices from the perspective of experts, who are routinely involved in the management of these patients. Moreover, the level of knowledge of IBD patients in developing countries such as Iran is significantly lower compared with their peers in developed regions. This then leads to undesirable consequences such as late diagnosis (Rezailashkajani et al., 2006). Owing to the importance of self-empowerment in patients with IBD and identifying informational and supportive needs and sources of obtaining information, the present study was designed to fill this gap via a Delphi consensus study.

## 2. Methods

### 2.1. Study design and registration protocol

A Delphi consensus study was designed to identify informational and supportive needs and sources of information for patients with IBD. This research was approved by the Institutional Ethics Committee of Mashhad University of Medical Sciences (IR.MUMS.REC.1400.230).

### 2.2. Motivations for the choice of the Delphi methodology

**Figure 1:**
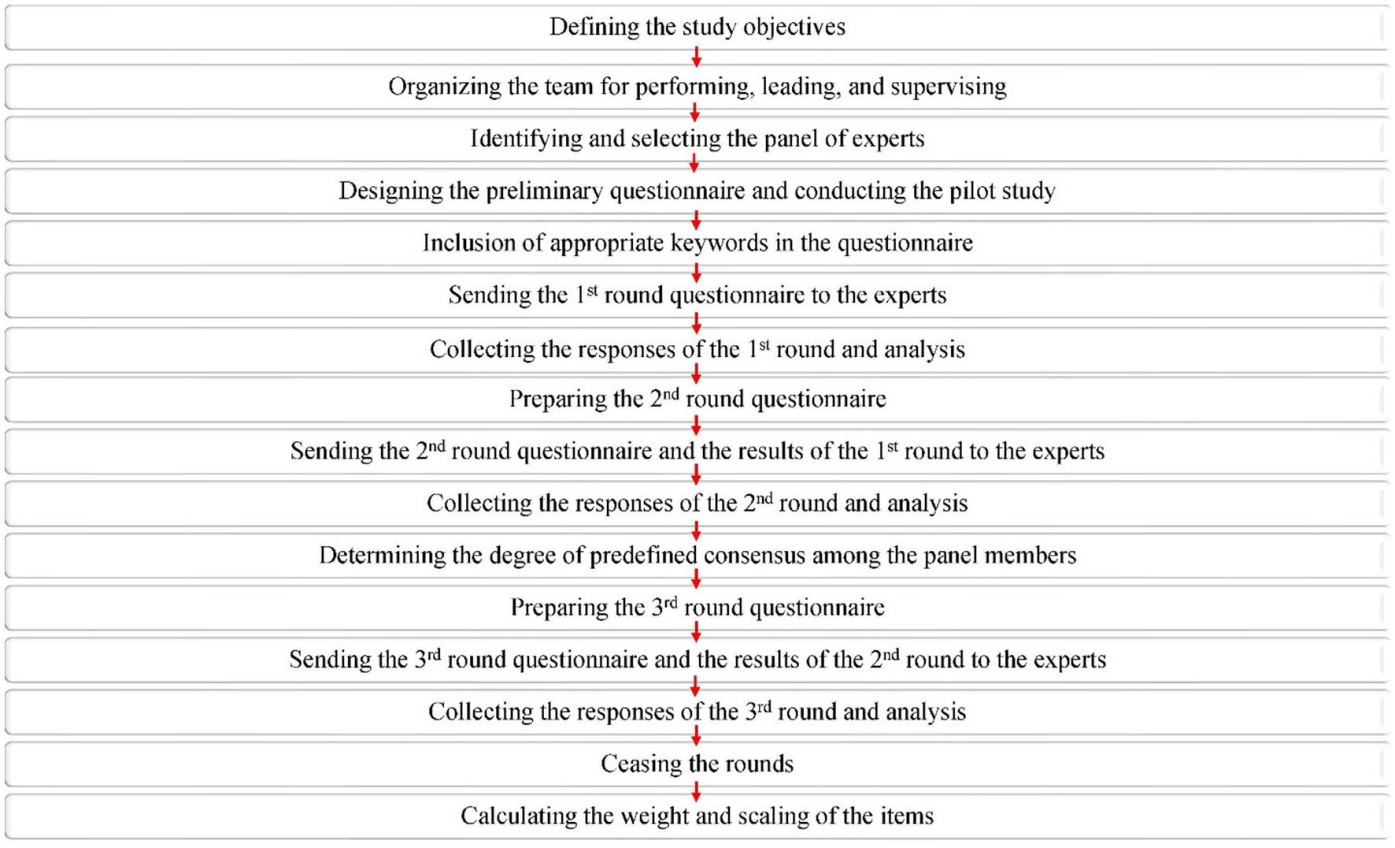
Main steps of Delphi consensus implemented in the present study.

Based on a scoping review, the current Delphi study is the second phase of investigations in the era of self-care aspects in patients with IBD (Norouzkhani et al., 2023). In the scoping review, important parameters such as informative, psychological, and supportive elements for IBD patients were extracted from the literature and reported. Owing to the various opinions on disease diagnosis and management, formal group consensus methods can deliver objective and subjective judgments. In addition, formal group consensus methods include a wide range of knowledge and experience, interaction between members, and stimulating constructive debate. Therefore, the scientific research committee team identified parameters that need to be evaluated, scrutinized, ranked, and weighted specifically by experts. In this way, upcoming investigations, such as those of interventional procedures, are feasible based on expert-filtered data. To summarize, because the findings of the scoping review are the prerequisite for conducting the next phase, Delphi consensus is the option of choice to integrate diverse viewpoints from experts in the field. The main steps of the Delphi approach are illustrated in Figure 1.

### 2.3. Research questions

The current study aims to seek answers to the following questions:

What is the experts' opinion on the informational needs of patients with IBD?
What is the experts' opinion on the supportive needs of patients with IBD?
What is the experts' opinion on sources for obtaining information on patients with IBD?

### 2.4. Identification and selection of experts

A steering committee consisting of experts in the field of IBD was identified and selected. They were responsible for performing, leading, and supervising all the research steps. These experts were in well known national specialists in the field of IBD. This team also defined certain criteria, primarily based on the regulations of the European Food Safety Authority (Authority, 2014), for selecting experts who were responsible for responding to the questionnaires. These inclusion criteria were years/type of experience, vocational qualifications, related references, publications, awards, conference presentations, academic qualifications, and teaching experiences. Other criteria, such as expressing judgments and experiences of risk assessment, were also considered. A steering committee first assessed the feasibility of the types of specialty for responding to the questionnaires and then attempted to identify them. Main national experts in the field of IBD were mapped according to the existing databases/literature/knowledge or those with the most relevant publications in this area through Internet searches. Even those with opposing views were invited. At this point, their CVs were requested if they were not found in the public database. Those experts who did not respond to the questionnaires after 14 days were excluded from the study. All the invited experts were asked to sign a form informing them about the study's subject and objectives, its duration, and approximate round numbers to show their agreement to participate.

In the current study, two sampling methods were used to establish the panel of experts. Initially, purposive sampling was utilized to select the first line of experts based on defined criteria. Then, snowball sampling was used to accelerate the process of finding experts and increase the number of panel members. First-line experts were asked to introduce other experts in accordance with the defined criteria. This method of selecting panel members was used because the researchers' committee had no precise information about their expertise, which significantly affected the study's outcome. Furthermore, because experts in a specific field usually knew each other well in the context of a scientific community, more experts were found in a shorter period of time. Indeed, experts communicate with each other more easily based on previous familiarity, and hence, they accept participation and membership in the panel more readily compared with invitations from the researchers' committee. The average age of the experts was 44.89 ± 6.44 years, and 56.14% of them (*n* = 32) were men. All of them were academics in universities and research institutes and were gastroenterologists (*n* = 24, 42.11%), psychologists/psychiatrists (*n* = 16, 28.07%), or nurses (*n* = 17, 29.82%). The total years of experience of the experts in the field of IBD were 15.49 ± 5.58 years. Table 1 depicts the number of panel members, their field of specialty, and the duration of their work experiences.

**Table 1:** Characteristics of panel members.

**Specialty**	***N***	**Duration of work experiences (years)**		
		<**10**	**10–15**	>**15**
Gastroenterology and hepatology (academic staff)	24	3	6	15
Psychiatry or clinical psychology (academic staff)	16	3	8	5
Nurse	17	2	5	10
Total	57	8	19	30

### 2.5. Design of the preliminary questionnaire and implementation of the pilot study

Our scoping review (Norouzkhani et al., 2023) identified important items in the informational needs, supportive needs, and sources of obtaining information. After omitting duplicates, 56 items in information needs, 36 items in supportive needs, and 36 items in sources of obtaining information were identified. Based on the retrieved items, specific questions were designed for each section, ultimately leading to a questionnaire with both open- and close-ended questions. The questionnaire was sent to four experts to find any possible pitfalls and misunderstandings within the questions. These experts were selected from three different provinces (Tehran, Khorasan Razavi, and Mazandaran). They discussed all the questions in the preliminary questionnaire and decided to replace some of them with more understandable questions with more suitable keywords if necessary. The experts proved the validity of the questionnaire and its content validity upon reaching a common understanding of the questions in line with the subject of the study. To check the reliability, Cronbach's alpha was calculated. Moreover, a test-retest examination was conducted for the questionnaires of the first and second rounds. As indicated in Tables 2–4, a 5-point Likert scale was defined, including one score for very low, two scores for low, three scores for moderate, four scores for high, and five scores for very high importance. In each section, the mean score was calculated for every question based on the received scores from all the experts, and this mean was considered for assigning the item to the low (<3) or high importance (≥3) category. If the question gained a high score, which means high importance, it was included in the next round of the questionnaire. Otherwise, it was omitted.

**Table 2:** First round questions of Information needs of patients with inflammatory bowel disease.

**In your opinion, how important are these information in educating and meeting the information needs of patients with inflammatory bowel disease?**						
		**Importance range**				
		**Very low (1)**	**Low (2)**	**Moderate (3)**	**High (4)**	**Very high (5)**
1	“General information about inflammatory bowel diseases”					
2	“Etiology”					
3	“Information on epidemiological results and pathogenesis”					
4	“Disease course and progress”					
5	“Clinical symptoms/appearance”					
6	“Defecation information”					
7	“Information on the anatomy/physiology of the digestive system”					
8	“Information on diagnostic methods including (types of diagnostic methods, risk of diagnostic methods and information on the interpretation of diagnostic tests)”					
9	“Prognostic information/long-term outcomes”					
10	“Information on risk factors/disease flare-up”					
11	“Prevention from recurrence and disease control”					
12	“Required actions during recurrence”					
13	“Extra-intestinal appearance/disease complications”					
14	“Cancer information”					
15	“Infection risk information”					
16	“Information related to co-morbidities and its management”					
17	“Information related to the mortality of inflammatory bowel disease”					
18	“Treatment and its side effects”					
19	“Medications and their side effects”					
20	“New research information and progress in inflammatory bowel diseases”					
21	“Participating information in research studies”					
22	“Adherence to medications”					
23	“Surgery information”					
24	“Information on non-pharmacological treatments”					
25	“Information on alternative and complementary medicine”					
26	“Information about COVID.19”					
		**Importance range**				
27	“Vaccination information”					
28	“Lifestyle and daily life information”					
29	“Information risky behaviors such as smoking” in educating and meeting the information needs of patients					
30	“Nutrition information”					
31	“Nutritional deficiency information”					
32	“Nutritional supplement information”					
33	“Enteral nutrition information”					
34	“Exercise or physical activity information”					
35	“Rehabilitation information”					
36	“Travel information”					
37	“Pain management and related symptoms”					
38	“Disease management and self-care information including (adjustment of medication according to conditions, individual patient follow-up plan, empowerment for self-management in relapse, home remedies, ...)”					
39	“Coping and living inflammatory bowel disease”					
40	“Information about social communication aspects”					
41	“Stories and experiences about dealing with the disease of other patients”					
42	“Disease control and struggling against stress and psychological issues”					
43	“Information on quality of life”					
44	“Information on religious and spiritual issues to deal with the disease”					
45	“Information related to gynecological issues”					
46	“Information about sexual relationship”					
47	“Information related to heredity/genetic information/microbiome”					
48	“Informing to the family or any other influential people”					
49	“Information related to the family (matters affecting the patient's family, effective training for the patient's family)”					
		**Importance range**				
50	“Information about work-related issues”					
51	“Interventions for sanitary and preventive care”					
52	“Social-health resource information”					
53	“Information about Hospitals/physicians”					
54	“Information related to when to see a doctor in case of symptoms emergence”					
55	“Information on legal and political aspects”					
56	“Information about insurance coverage/financial support/treatment and drug costs”					

**Table 3:** First round questions of supportive needs of patients with inflammatory bowel disease.

**In your opinion, how important are these items in supporting patients with inflammatory bowel disease?**						
		**Importance range**				
		**Very low (1)**	**Low (2)**	**Moderate (3)**	**High (4)**	**Very high (5)**
1	“Supporting psychological/emotional needs”					
2	“Screening and psychological assessment”					
3	“Psychological support (counseling, psychotherapeutic intervention and follow-up)”					
4	“Coping skills with the disease”					
5	“Psychological self-care”					
6	“Management of physical needs”					
7	“Addressing the concerns of life and death and spiritual issues”					
8	“Educating the patient the ability to obtain information, including recommending educational resources”					
9	“Educating patient”					
10	“Providing appropriate information (clear, structured and factual information and providing information at the right time)”					
11	“Educating/informing family, friends and colleagues”					
12	“Attention to the patient's family or caregivers”					
13	“Social health support systems and support groups”					
14	“Participation of the family/partner of the patient/caregiver”					
15	“Acquiring social skills”					
16	“Advocacy to marital intimacy”					
17	“Advocacy of the needs and problems related to gestation and fertility”					
18	“Access to health care”					
19	“Facilities (availability of care facilities, availability of toilets in clinics, no need to share a room with others, quality of health in hospitals, quick visit in case of recurrence and fast diagnosis and regular follow-up)”					
20	“Legal support”					
21	“Financial support”					
22	“Occupational support”					
23	“Supporting activities of daily living”					
24	“Disease management or self-care”					
25	“Support from nutritionists”					
26	“Support from psychologists/psychiatric specialists”					
27	“Multidisciplinary care services/holistic approach”					
		**Importance range**				
28	“Shared/patient-centered decision-making approaches”					
29	“Technological support”					
30	“Information sharing and good coordination between gastroenterologists, other professionals and patients”					
31	“Support and appropriate interaction between doctor and patient”					
32	“Easy access and contact with health workers and specialists”					
33	“Urgent advice (by phone, or clinic appointments)”					
34	“Monitoring and follow-up of the patient's condition”					
35	“Advocacy and support for experiencing cognitive needs such as memory loss”					
36	“Future support needs such as attention and support for transition needs”					

**Table 4:** First round questions of information sources and methods needs of patients with inflammatory bowel disease.

**In your opinion, how important and useful are these sources of obtaining information useful for patients with inflammatory bowel disease?**						
		**Importance range**				
		**Very low (1)**	**Low (2)**	**Moderate (3)**	**High (4)**	**Very high (5)**
1	“Health professionals team”					
2	“Gastroenterologist”					
3	“Family physician/general practitioner”					
4	“Nurse”					
5	“Nutritionists”					
6	“Physiotherapist”					
7	“Psychiatrists/psychologists”					
8	“Pharmacist”					
9	“Surgeon”					
10	“Traditional medicine or complementary medicine physicians”					
11	“Inflammatory bowel diseases hospitals/clinics”					
12	“Health network”					
13	“Brochure or pamphlet”					
14	“Magazines and newspapers or the press”					
15	“Materials prepared by the physician”					
16	“Tv/radio”					
17	“Educational video clip”					
18	“Surfing the Internet”					
19	“Websites”					
20	“Social networks”					
21	“Applications of mobile phones”					
22	“Internet-based sources”					
23	“Telephone-based information services”					
24	“Email”					
25	“Friends/acquaintances/family”					
26	“Personal/other patients' experiences”					
27	“Insurance”					
28	“Support and advisory services/associations for patients with inflammatory bowel disease”					
29	“Travel counseling centers”					
30	“Legal representation of patients” in obtaining information					
31	“Pharmaceutical companies or research institutes”					
		**Importance range**				
32	“Scientific and medical papers and journals”					
33	“Summaries of conference articles”					
34	“Medical books”					
35	“Medical encyclopedia”					
36	“Summary of scientific researches”					

Experts who participated had no direct interaction with each other, and data were exchanged via an Internet-based platform without physical contact. Generally, in this method, experts were asked to send their responses and any possible comments on consecutive questionnaires according to the cumulative feedback from the previous round. The feedback helped the experts to reevaluate, modify, or expand the comments (Windle, 2004). The promising advantage of such an approach is that it ensures anonymity for the participants. Such anonymity ensures that no specific expert would have a dominant effect on others' opinions (Dalkey, 1969; Landeta, 2006), allowing all individuals to have the same opportunity to express their own opinions. This way, it facilitates the free expression of ideas and helps acquire sufficient insight and knowledge in the field (Walker and Selfe, 1996; Turoff and Linstone, 2002; Ali, 2005).

A web-based platform was used for sending the first round of questionnaires. An analysis of the responses collected from the first round formed the basis for the preparation of the second round questionnaire. Based on the results from the first round, five questions out of 56 in the information needs section, one out of 36 in the supportive needs section, and seven out of 36 in the information sources section had scores lower than the mean. Hence, these questions were regarded as having low importance and omitted from the questionnaire. Moreover, experts agreed to add one item (fasting) to the information needs section and another one (acquiring psychological skills) to the supportive needs section. They believed that these two are effective in recognizing information and supportive needs in IBD patients. The second round of questionnaires and the results of the first round were sent to the experts via the web-based platform. The experts were informed in detail about the changes. Experts' comments were collected in the second round and combined to provide scoring for each question. Based on the findings extracted from the second round, a third round of questionnaires was designed. Seven questions out of 52 in the information needs section, three out of 36 in the supportive needs section, and seven out of 29 in the information sources section were found to have scored lower (<3) than the mean. These questions were regarded as having low importance and omitted from the questionnaire. At this step, no further items were proposed for adding to the questionnaire. After sending the new questionnaire to the experts and collecting their comments, they were subjected to analysis.

### 2.6. Ceasing the rounds

After the third round, analysis of the responses showed that the scores for all the questions were higher than the mean. The experts proposed no new statements at this stage. Furthermore, the results of all three rounds of this Delphi approach showed that experts' consensus had been reached for the following reasons: (1) No statement was omitted or added in the third round, (2) given that the number of respondents was more than 10 individuals and the Kendall coefficients were 0.330, 0.344, and 0.325 in three sections at the third round, a completely meaningful condition was deduced, and (3) there was a slight difference between the second and third rounds without significant growth in the Kendall coefficient.

### 2.7. Calculation of the weight and scales of the items

After finalizing the identification of important items in three sections, the weight and scale of each item were determined based on the scores assigned by the experts at the end of round three.

## 3. Results

In the present study, various important items were identified in the areas of informational needs, supportive needs, and sources of obtaining information using the Delphi consensus for patients with IBD and were presented from the viewpoints of experts using the consensus. Based on previous findings (Norouzkhani et al., 2023), a preliminary questionnaire was designed (Tables 2–4). In a scoping review, we previously identified informational needs, information resource, and supportive needs, as well as psychological needs, of IBD patients based on the Daudt methodological framework (Norouzkhani et al., 2023). After defining the research questions according to the four sections mentioned, all types of studies that were conducted in patients with IBD and ≥18 years of age were considered without any restrictions in the language or settings. A single consensus strategy based on the inclusion and exclusion criteria was defined, and electronic databases were extensively searched from January 2000 to April 2022. After omitting duplicates and screening the titles, the abstracts of the remaining papers were separately scrutinized by two independent experts. To ensure the similarity of the decisions made by these two experts on the inclusion and exclusion of the papers, 10% of them were checked by a third expert. At the next stage, full texts were assessed, and any disagreements were resolved by a third party. According to the guidelines for conducting the scoping reviews (Peters et al., 2015), there was no need to appraise the methodological quality or risk of bias in the included papers.

The resulting questionnaires were delivered to 79 other experts in the first round, and only 57 of them answered. There was a non-normal distribution among the collected parameters. As shown in Table 5, the Cronbach alpha was calculated at 0.760, which shows the reliability of the questionnaire. A total of 13 questions out of 128 were omitted from the questionnaire due to their low importance based on the scores. In terms of informational needs, items that were omitted were “participating information in research studies,” “rehabilitation information,” “information about social communication aspects,” “social-health resource information,” and “information on legal and political aspects.” Only the item “advocacy and support for experiencing cognitive needs such as memory loss” was omitted from the supportive needs section.

**Table 5:** Cronbach's alpha value.

	**Questionnaire elements**	**Number of items**	**Cronbach's alpha**
1st round	Information needs	56	0.704
	Supportive needs	36	0.710
	Information sources	36	0.705
	Total	128	0.760

Regarding the information sources section, “physiotherapist,” “health network,” “email,” “friends/acquaintances/family,” “travel counseling centers,” “legal representation of patients in obtaining information,” and “medical encyclopedia” were removed. Meanwhile, two further items (fasting and acquiring psychological skills) were added to the questionnaire. After analyzing the responses from the first round, the Kendall rank correlation coefficient was used to determine the degree of convergence. The Kendall coefficient of 0.354 for information needs, 0.252 for supportive needs, and 0.353 for sources of obtaining information showed ~35%, 25%, and 35% convergence between the experts' viewpoints, respectively.

The number of questions was decreased to 117 by making the required changes. Analyzing the results of the second round of the questionnaire revealed that 17 questions indicated low importance based on their scores. The lack of any further items to be added to the questionnaire indicated that the current version had covered all aspects of the study objectives. In the second round, the Kendall coefficient was 0.343 for information needs, 0.310 for supportive needs, and 0.363 for sources of obtaining information, showing ~34%, 31%, and 36% convergence between the experts' viewpoints, respectively. Although the Kendall coefficient was meaningful at this stage, this does not provide sufficient evidence to cease the Delphi approach in the second round because there were still some questions of low importance in the second questionnaire, and therefore, the experts had not reached a consensus.

Omitting 17 indices of low importance produced a questionnaire with 100 questions. All the questions in the third round had a score equal to or greater than the mean. It was deduced that all the remaining items were of high importance. The Kendall coefficient was calculated to be 0.344 for information needs, 0.330 for supportive needs, and 0.325 for sources of obtaining information, meaning that there was ~34%, 33%, and 32% convergence between the experts' viewpoints, respectively. Similarly, in the second round, no other items were proposed by the experts, indicating that the current ones had encompassed all aspects of the study. The criteria for ceasing the rounds were provided, as there were no omissions or additions for any other items, and the difference in the Kendall coefficients between the second and the third rounds was not significant. Finalized items are provided in Tables 6–8 based on their weight and scaling.

**Table 6:** Approved items in information needs section after three rounds of Delphi consensus presenting in the order of weight and scaling.

**Priority**	**Index**
1	• Medications and their side effects • Pain management and related symptoms
2	• Clinical symptoms/appearance • Prevention from recurrence and disease control • Required actions during recurrence • Treatment and its side effects • Adherence to medications • Disease management and self care^*a*^
3	• General information about inflammatory bowel diseases • Defecation • Risk factors/disease flare-up
4	• Disease course and progress • Prognosis/long-term outcomes • Risky behaviors like smoking • Nutrition
5	• Information related to when to see a doctor in case of symptoms emergence
6	• Vaccination • Nutritional deficiency • Coping and living inflammatory bowel disease
7	• Extra-intestinal appearance/disease complications • Risk of infections • Co-morbidities and its management • Disease control and struggling against stress and psychological issues • Gynecological issues
8	• Non-pharmacological treatments • Exercise/physical activity • Stories and experiences about dealing with the disease of other patients • Quality of life • Informing to the family or any other influential people
9	• Religious fasting
10	• Cancer • COVID19 • Nutritional supplements
11	• Family^*b*^
12	• New research information and progress in inflammatory bowel diseases
13	• Interventions for sanitary and preventive care
14	• Etiology • Alternative and complementary medicine • Travel
15	• Hospitals/physicians
16	• Diagnostic methods^*c*^ • Mortality • Surgery • Sexual relationship

a Including medication dose proportionate to conditions, self-follow up program, empowerment in order to augment self-care in the case of disease recurrence, in-home therapy, and etc.

b Including important issues that affect the patients' family and useful educations for patients' family.

c Including different diagnostic methods and information to interpret the tests results.

**Table 7:** Approved items in supportive needs section after three rounds of Delphi consensus presenting in the order of weight and scaling.

**Priority**	**Index**
1	Coping skills with the disease
2	Supporting psychological/emotional needs Screening and psychological assessment Psychological support^a^ Psychological self-care Providing appropriate information^b^ Support and appropriate interaction between doctor and patient
3	Educating the patients Educating/informing the family, friends, and colleagues Participation of the family/partner of the patient/caregiver Advocacy of the needs and problems related to gestation and fertility Occupational support Disease management or self-care Multidisciplinary care services/holistic approach Urgent advice (by phone, or clinic appointments)^c^ Monitoring and follow-up of the patient's condition Psychological support^*d*^
4	Management of physical needs Attention to the patient's family or caregivers Advocacy to marital intimacy Shared/patient-centered decision-making approaches Information sharing and good coordination between gastroenterologists, other professionals and patients Easy access and contact with health workers and specialists
5	Educating the patient the ability to obtain information, including recommending educational resources Social health support systems and support groups Access to health care Facilities^e^ Financial support
6	Acquiring social skills Supporting activities of daily living Support from psychologists/psychiatric specialists Technological support
7	Support from nutritionists

a Including consultation, psychotherapy interventions, and follow up.

b Including clear, structured, and real information in appropriate time.

c These are delivered through phone calls or medical appointments.

d These include counteracting against stress, solving-problem skills, and capability to address conflicts between individuals.

e This means availability of care facilities, availability of toilets in clinics, no need to share the rooms with others, sanitation level in hospitals, and instant visits, fast diagnosis, and routine follow up in case of recurrence.

**Table 8:** Approved items in information sources and methods section after three rounds of Delphi consensus presenting in the order of weight and scaling.

**Priority**	**Index**
1	Gastroenterologist
2	Materials prepared by the physician
3	Health professionals team Applications of mobile phones
4	Psychiatrist/psychologist Surgeon Brochure/pamphlet Educational video clip Telephone-based information services
5	Nurse Nutritionist Personal/other patients experiences Support and advisory services/associations for patients with inflammatory bowel disease^*a*^
6	Family physician/general practitioner Inflammatory bowel disease hospitals/clinics Tv/radio Internet-based sources Scientific and medical papers and journals Summaries of conference articles
7	Websites Social networks Summary of scientific researches

a Association of inflammatory bowel disease patients.

## 4. Discussion

The main aim of the present study was to provide a comprehensive set of important items in the management of IBD in three sections: information needs, supportive needs, and sources of obtaining information for patients with IBD based on the experts in the field. These three sections constitute critical indices that patients with IBD need to control and manage the disease. Moreover, this study not only precisely discriminated important items from other ones but also allowed the classification of the important items via a rating system. In other words, this makes the differentiation of the most and least remarkable items among important items feasible. In our study, a 128-item questionnaire in the first round was optimized into a questionnaire with 100 items after three rounds of the Delphi consensus. The findings of this study were derived from the combined viewpoints of major stakeholders in the field of IBD, including gastroenterologists, psychiatrists/psychologists, and nurses, through a Delphi consensus approach. A convergence of 32–34% among experts' viewpoints was reached at the end of the process, indicating an efficient consensus process.

Identifying the needs of IBD patients in precise categories is beneficial for patients, physicians, and other healthcare professionals. Such information enables patients to manage the disease, alleviate relevant anxiety and worries, and improve their compliance. Otherwise, screening for some negative consequences of IBD, such as colorectal cancer, is underestimated by patients. Moreover, patients feel they have control over medical decisions, which causes a positive relationship with their physicians and healthcare professionals, which in turn makes patients feel less alienated. Moreover, this awareness reduces the upcoming complications that affect the three parties in terms of overcoming the barriers in formal and informal support, increasing the support intake from different resources, and directing the delivery of information to the patients in a more conducive and systematic manner.

Owing to internal (lack of control over bowel movements) and external stressors (access to restrooms), patients with IBD require specific supportive needs, which multi-professional teams should develop. One study identified instrumental support (disease-related information) and emotional support (discussing disease management). To support IBD patients, various strategies (behavioral, social, and emotional) were adopted to cope with disease conditions (Larsson et al., 2017). In our study, experts believe that “disease compatibility skills” would be of the highest priority regarding supportive needs.

There is a paucity of information regarding IBD among patients. Intriguingly, it was reported that patients with different profiles of demographic characteristics and clinical parameters have unique and clinically relevant information needs (Daher et al., 2019). Insufficient efforts in delivering specific domains of information to IBD patients may impede the identification of symptoms required for disease diagnosis. Indeed, information is a valuable element, and it can be regarded as a potentially important component that improves IBD outcomes (Pittet et al., 2016). However, most IBD patients believe that they did not receive important information about the disease in the first 2 months after diagnosis (Bernstein et al., 2011). Notably, disease duration affects the patients' knowledge. Those who are recently diagnosed may need different types of information compared with those with chronic illnesses (Bernstein et al., 2011).

IBD patients need information to manage the disease in their daily routine. This was referred to as “knowledge needs” in one study (Lesnovska et al., 2014) and was classified into three groups: those related to the disease course, those related to managing everyday life, and those difficult to understand and assimilate. This type of need has great variation, especially at the time of diagnosis and during relapse. “Medications and their side effects” and “pain management and related symptoms” were identified as the most important items in the information needs section of the present study. In one study on IBD patients from Greece, the main complaint was the lack of information about treatment. The study revealed that certain hurdles in some aspects of their lives, such as health-related social life, emotional status, and work productivity, were significantly affected (Viazis et al., 2013). Treatment (medical and surgical), clinical appearance, cancer, and mortality risks are the types of additional information needed by the patients (Catalán-Serra et al., 2015).

With respect to the sources of obtaining information, “gastroenterologists” were known as the major sources in our study. Another study reported that gastroenterologists, besides the Internet, were the most frequent sources of information 2 months after diagnosis. However, it was shown that only 45% of patients were very satisfied with the information they received at the time of diagnosis (Bernstein et al., 2011). In third place, general practitioners were known as sources for obtaining information another study. Once more, only about half of the patients claimed that the gastroenterologists covered their information needs. Furthermore, it should be noted that the Internet was useful for young patients and those with a high level of literacy (Catalán-Serra et al., 2015).

Both the mental and physical health of IBD patients are impaired, according to the findings from one study, which showed that general health perceptions were below the critical value in 40% of patients. This demonstrates the importance and divergence of needs among IBD patients (Casellas et al., 2020). In a survey to identify the needs of young adults with IBD, psychological needs and daily living needs were presented as the most and least common ones (Cho et al., 2018). Because the burden of psychological distress was found to be concerning in such patients, point-of-care screening and interventions should be considered initially in the context of biopsychological care (Moon et al., 2020).

Clinical conditions of IBD patients, such as laboratory findings, activity parameters, and endoscopic examinations, traditionally form the basis of daily practice and care plans, such as the type of medications, frequency of visits, and referral to another specialist (Sainsbury and Heatley, 2005; Sajadinejad et al., 2012; Moradkhani et al., 2013; Williet et al., 2014). Although quality of life is improved by such an objective evaluation of the disease, some subjective aspects based on patients' characteristics such as personality, expectations, family framework, and social issues are also determined (Casellas et al., 2020). Nowadays, holistic and personalized medicine have become novel features in therapeutic approaches that alter the model of care for chronic diseases like IBD (Kennedy and Rogers, 2002; Baars et al., 2010). In line with this, the empowerment of the patients, their involvement in disease management, and incorporating their opinions into clinical decisions seem vital (O'Connor et al., 2013; Rettke et al., 2013). Implementing these approaches improves quality of life and creates satisfaction in patients regarding the kind of care and treatment they receive (Barlow et al., 2010; O'Connor et al., 2014). In a scoping review, the nature and extent of the research evidence were published for IBD patients across three life cycles. Scrutinizing the main needs of children, adolescents, and adults showed the value of the involvement of the patient and healthcare providers through supporting and promoting engagement. Moreover, such interventions were advised to be organized from a multidisciplinary perspective (Volpato et al., 2021).

Empowerment of IBD patients significantly contributes to rehabilitation programs and helps them handle the long-term consequences of the disease and manage their health status more efficiently by obtaining better outcomes (Small et al., 2013). Empowerment, as a complicated experience of personal modifications in life values and priorities, is classified at individual, organizational, and community levels (Aujoulat et al., 2007). In one study, the key aspects of empowerment in IBD patients were reported to be social interaction skills and disease-specific health literacy (Zare et al., 2020). Interactions with others in the form of communicating with optimistic people, establishing family and friendly entertainment plans, and having relations with peers improve the mental status of patients and are considered an efficient approach for controlling psychological situations that trigger IBD flare-ups. The ability to ask for support is another aspect of such interactions, which can be substantiated by asking physicians to speak to the relatives of the patients for support, meet coworkers/bosses about the disease, and demand reliable information in web-based tools from valid sources (Zare et al., 2020).

The Internet is not only a learning source for IBD patients but also a substrate for communication. Patients who use the Internet are young, more educated, and sicker (Angelucci et al., 2009). While the Internet delivers a considerable amount of information to IBD patients (Cima et al., 2007), it affects the relationship between patients and the physicians. It should be noted that information derived from the Internet is usually unregulated and unfiltered, and this may lead to confusion and mislead people into making poor choices. Some Internet-based applications, or social media applications, allow for exchanging ideas and facilitate interpersonal interaction (Kaplan and Haenlein, 2010), eventually resulting in patient empowerment (Flisher, 2010). In addition, some applications are designed for use on smartphones and mobile tablets and help patients with symptom tracking and self-management. Some other applications are equipped to print or email reports to the physicians. However, some limitations, such as a lack of clarity in the qualifications of the providers, production of design and develop disease management products without consultation with IBD care providers, and the absence of validated measures of disease severity, restrain their use (Fortinsky et al., 2012).

To meet a wide range of needs that are considered important by patients, providing reliable applications would be an outstanding help. Although patients can access huge amounts of data through the Internet, social media, and support groups, they prefer to receive information about their disease mainly from physicians. However, transferring all the required information from physicians to patients through traditional verbal communication appears to be impractical. To overcome this barrier, written information, such as brochures or websites with user-friendly interfaces, is an appropriate alternative that supplements physician–patient consultations and provides a higher level of detailed information (Bernstein et al., 2011).

Reaching a high level of agreement is one of the strengths of the present study. This shows the validity of the consensus process that was obtained from the opinions of health experts from different fields. The inclusion of patients' preferences in a multidisciplinary way is necessary for clinical care.

### 4.1. Limitations

The findings of this study may not be generalizable to all IBD patients due to differences in the characteristics of IBD patients (prevalence and severity of the disease). All the invited experts were from the same country, and their opinions may differ from those of their peers in other regions. Furthermore, it is logistically impossible to gather all the experts in the field from different specialties. Distribution of experts with sufficient skill and expertise in managing IBD patients are not homogenous between regions and countries. Patients are not homogenous between regions and countries. Health infrastructures and facilities, such as centers specifically organized to support IBD patients in terms of medications and other needs, are not equally available between high- and middle-income countries. The comprehensive nature of care in chronic diseases such as IBD requires different healthcare providers, such as nutritionists, gynecologists, radiologists, general practitioners, stoma therapists, rheumatologists, physician assistants, pharmacists, and immunologists, to be involved in delivering diverse information to patients. For instance, mucosal immunologists, who are among the most important experts with critical roles in the differential diagnosis of various forms of IBD (CD and UC), were not present in our study. All these factors, in our opinion, may limit the generalizability of the findings of the current study.

## 5. Conclusion

The present study identified 100 items across three categories: supportive needs, sources of obtaining information, and the specific informational needs of IBD patients, as identified by experts using a Delphi-based methodology. Properly educating IBD patients based on verified needs can result in decreased stress levels, improved treatment adherence, and enhanced disease control and management. Although we believe that these questionnaires are useful for national patients in delivering certain information and meeting some needs, future studies should be conducted with the inclusion of a broader range of experts from both basic and clinical specialties and with the participation of different centers from different regions and countries to identify specific and more generalizable needs for IBD patients.

## Data availability statement

The original contributions presented in the study are included in the article/supplementary material, further inquiries can be directed to the corresponding author.

## Ethics statement

The studies involving human participants were reviewed and approved by Mashhad University of Medical Sciences (IR.MUMS.REC. 1400.230). The patients/participants provided their written informed consent to participate in this study.

## Author contributions

NN and HT conceived the original idea. NN carried out the experiment and wrote the manuscript with support from HT and MF. AB, MF, and JS carried out the experiment and aided in interpreting the results. SE and AB helped supervise the project. HT supervised the project. All authors reviewed the results and approved the final version of the manuscript.
